# Immunopurification of Pathological Prion Protein Aggregates

**DOI:** 10.1371/journal.pone.0007816

**Published:** 2009-11-12

**Authors:** Emiliano Biasini, Laura Tapella, Susanna Mantovani, Matteo Stravalaci, Marco Gobbi, David A. Harris, Roberto Chiesa

**Affiliations:** 1 Dulbecco Telethon Institute, Milan, Italy; 2 Department of Neuroscience, Mario Negri Institute for Pharmacological Research, Milan, Italy; 3 Department of Biochemistry and Molecular Pharmacology, Mario Negri Institute for Pharmacological Research, Milan, Italy; 4 Department of Cell Biology and Physiology, Washington University School of Medicine, St. Louis, Missouri, United States of America; Brigham and Women's Hospital/Harvard Medical School, United States of America

## Abstract

**Background:**

Prion diseases are fatal neurodegenerative disorders that can arise sporadically, be genetically inherited or acquired through infection. The key event in these diseases is misfolding of the cellular prion protein (PrP^C^) into a pathogenic isoform that is rich in β-sheet structure. This conformational change may result in the formation of PrP^Sc^, the prion isoform of PrP, which propagates itself by imprinting its aberrant conformation onto PrP^C^ molecules. A great deal of effort has been devoted to developing protocols for purifying PrP^Sc^ for structural studies, and testing its biological properties. Most procedures rely on protease digestion, allowing efficient purification of PrP27-30, the protease-resistant core of PrP^Sc^. However, protease treatment cannot be used to isolate abnormal forms of PrP lacking conventional protease resistance, such as those found in several genetic and atypical sporadic cases.

**Principal Findings:**

We developed a method for purifying pathological PrP molecules based on sequential centrifugation and immunoprecipitation with a monoclonal antibody selective for aggregated PrP. With this procedure we purified full-length PrP^Sc^ and mutant PrP aggregates at electrophoretic homogeneity. PrP^Sc^ purified from prion-infected mice was able to seed misfolding of PrP^C^ in a protein misfolding cyclic amplification reaction, and mutant PrP aggregates from transgenic mice were toxic to cultured neurons.

**Significance:**

The immunopurification protocol described here isolates biologically active forms of aggregated PrP. These preparations may be useful for investigating the structural and chemico-physical properties of infectious and neurotoxic PrP aggregates.

## Introduction

Prion diseases are fatal degenerative disorders of the central nervous system (CNS) that can arise sporadically, be genetically inherited due to mutations in the gene encoding the prion protein (PrP), or acquired through infection [1]. The majority of prion diseases involve CNS accumulation of PrP^Sc^, an abnormally folded form of the cellular prion protein (PrP^C^), which propagates itself by seeding conformational conversion of PrP^C^ substrate molecules [2], [3].

PrP^Sc^ and PrP^C^ have distinct biophysical and biochemical properties. PrP^Sc^ is rich in β-sheet structure, insoluble in mild detergents, and partially resistant to digestion with proteinase-K (PK), yielding a N-terminal truncated fragment of 27–30 kDa (PrP27-30) [4]–[6]. In contrast, PrP^C^ has a predominant α-helix structure [7], is soluble in detergents and PK-sensitive.

PrP^Sc^ is pathognomonic of prion infection; however, it may not be the proximate cause of neurodegeneration [8]. Several genetic prion diseases, in fact, develop in the absence of protease-resistant PrP or in the presence of other abnormal forms of the protein, and are not transmissible to laboratory animals [9]–[13]. Some sporadic prion diseases have also been described that do not have PK-resistant PrP in the CNS [14], [15], reinforcing the idea that PrP refolding into PrP^Sc^ is not required to induce neurodegeneration.

Experiments in transgenic (Tg) mice support the contention that pathogenicity and infectivity are independent properties of misfolded PrP, attributable to different conformational states of the protein. Tg(PG14) mice carrying the mouse PrP homologue of a 9-octapeptide repeat insertion linked to a genetic prion disease develop a progressive neurological illness with massive apoptosis of cerebellar granule neurons [16], [17]. These mice synthesize a misfolded form of mutant PrP in their brains that shows a high tendency to aggregate but has considerably less protease resistance than conventional PrP^Sc^, and is not infectious [17]–[19]. When inoculated with Rocky Mountain Laboratory (RML) prions, however, Tg(PG14) mice accumulate a form of PG14 PrP that is easily distinguished from the one produced in spontaneously ill mice, because it is highly PK-resistant, infectious in animal bioassay and able to seed PrP^C^ misfolding in a protein misfolding cyclic amplification (PMCA) reaction [18], [19]. It is still not clear what structural features distinguish infectious PG14 PrP from the non-infectious form of the protein [19].

A number of methods have been developed for purifying PrP^Sc^ from prion-infected animals for biological and structural analyses [6], [20]–[22]. Commonly used procedures are based on sequential centrifugation of detergent brain extracts to concentrate insoluble PrP^Sc^ molecules, and incubation with high concentrations of PK to digest PrP^C^ and other proteins, yielding 60–90% pure PrP27-30 preparations. These protocols cannot be used to purify pathological PrP species lacking conventional PK resistance.

Here we describe a method for purifying aggregates of misfolded PrP, based on immunoprecipitation with a monoclonal antibody that recognizes structural epitopes common to both infectious and non-infectious PrP [23]–[25]. This procedure can be used to isolate aggregated full-length PrP^Sc^ molecules from prion-infected mice, as well as neurotoxic PrP aggregates that accumulate in the brains of Tg mice expressing pathogenic PrP mutations. PrP preparations obtained with this method are highly pure, and can be used for structural and physicochemical studies.

## Results

### Monoclonal Antibody 15B3 Reacts with Semi-Purified PG14 PrP Aggregates

**Figure 1:**
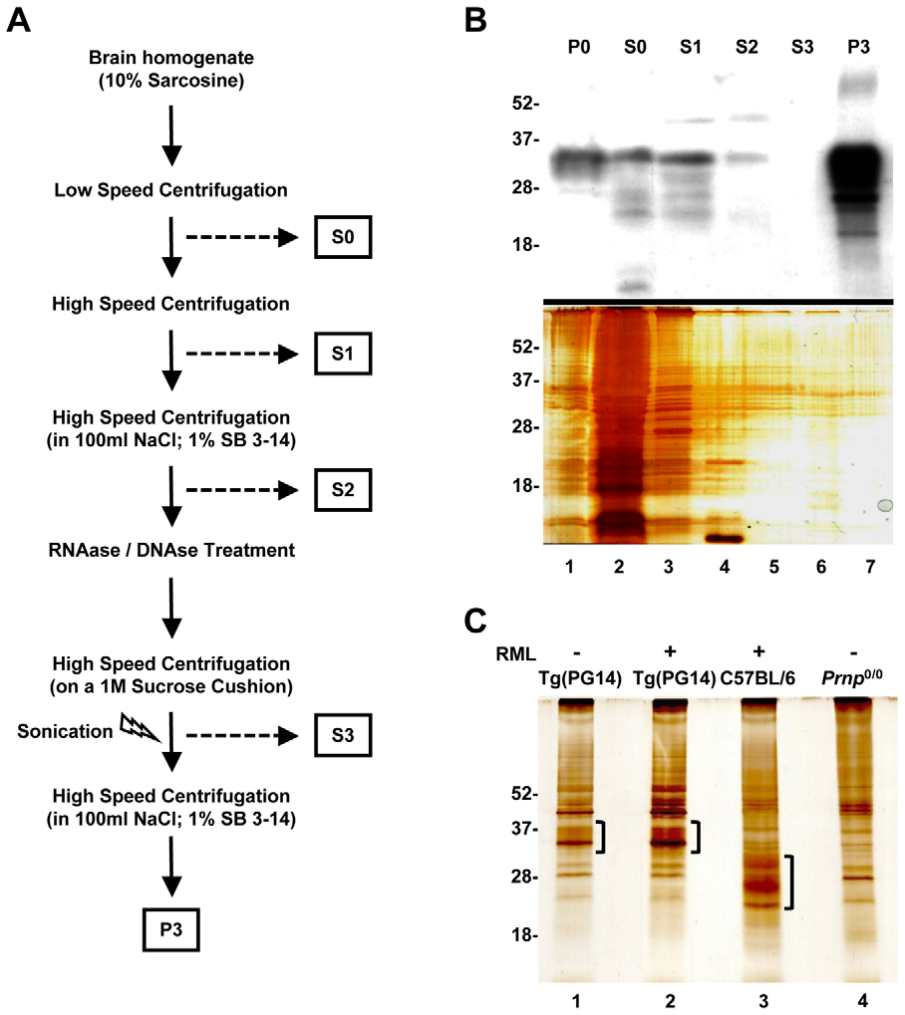
Aggregates of PG14 PrP can be enriched by sequential centrifugation. Brain homogenates from Tg(PG14) mice were fractionated by the scheme shown in (A). (B) Proteins in the different fractions were Western blotted using anti-PrP antibody 3F4 (top panel), or separated by SDS-PAGE and visualized by silver staining (bottom panel). (C) Proteins in the P3 fractions isolated from un-inoculated (−) or RML-infected (+) mouse brains were separated by SDS-PAGE and visualized by silver staining. Brackets indicate the protein bands corresponding to PrP (lanes 1–3). Molecular size markers are given in kilo-daltons. For this and all subsequent figures, results were reproduced in at least three independent experiments, each using different mouse brains.

A common procedure for purifying PrP^Sc^ from prion-infected brains consists of a series of sequential centrifugation gradually enriching insoluble PrP [20], [22] (Fig. 1A). This protocol is commonly used to isolate PrP^Sc^ from infected Syrian hamsters, which accumulate high levels of insoluble PrP in their brains [6], [26].

We used sequential centrifugation to purify insoluble mutant PrP molecules from the brains of Tg(PG14) mice. To evaluate the efficiency of purification, we assayed PrP in the different fractions by Western blot, and compared the amount to the total amount of proteins in the corresponding fraction, visualized by silver staining (Fig. 1B and C). Insoluble PG14 PrP was highly enriched in the final pellet (P3), which contained approximately 60% of total PrP (Fig. 1B). However, PrP still accounted for a small proportion of the proteins in this fraction (Fig. 1C, lane 1). Similar low-purity preparations were obtained when sequential centrifugation was used to isolate PrP^Sc^ from the brains of RML-infected Tg(PG14) or C57BL/6 mice (Fig. 1C, lanes 2 and 3). The P3 fraction from the brains of PrP knockout mice (*Prnp* ^0/0^) showed a similar pattern of bands (with the exception of the PrP-specific bands) (Fig. 1C, lane 4), confirming that the P3 pellet contained a number of detergent-insoluble proteins besides PrP.

**Figure 2:**
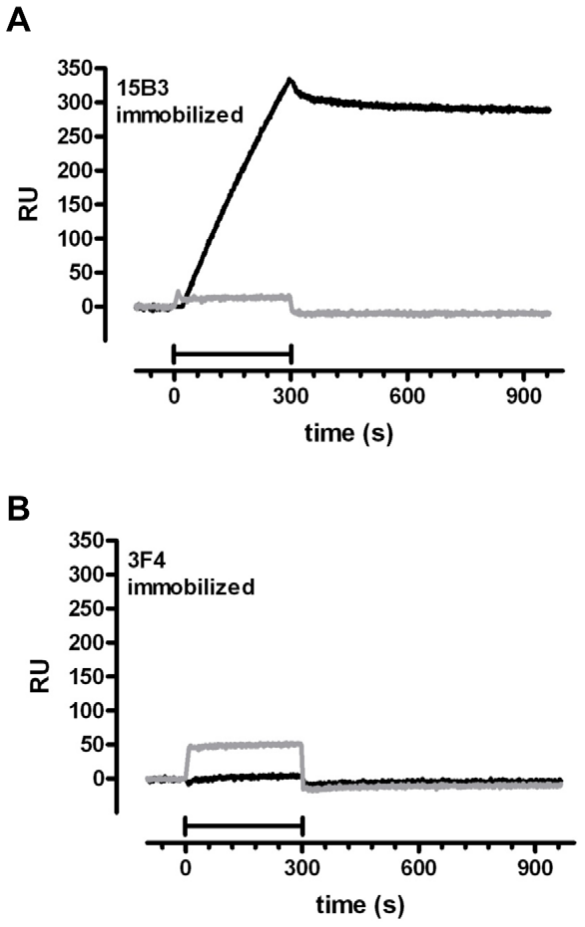
Surface plasmon resonance shows that monoclonal antibody 15B3 selectively captures PG14 PrP aggregates from P3 fractions. Fractions P3 prepared from whole brains of Tg(PG14) or *Prnp* ^0/0^ mice were perfused for 5 min (bars) over the sensor surface of SPR chips on which antibody 15B3 (A) or 3F4 (B) had been immobilized. The sensorgrams (time course of the SPR signal in Resonance Units, RU) refer to the specific binding to the antibody (binding was negligible on sensor surfaces without antibody). Significant binding was detected when P3 fractions containing PG14 PrP (black line) were flowed over the 15B3-coated chip (A), but not on the 3F4-coated chip (B). No binding was observed when a P3 fraction from *Prnp* ^0/0^ mice was analyzed (gray line).

Monoclonal antibody15B3 reacts selectively with PrP^Sc^ [23], as well as with non-infectious aggregates of mutant PrP [24], [25]. We used this antibody to immunopurify PG14 PrP. To test whether 15B3 could selectively capture the protein from P3 fractions, we used surface plasmon resonance (SPR). P3 fractions from Tg(PG14) or *Prnp* ^0/0^ mice were injected onto SPR sensor chips in which monoclonal antibody 15B3 or 3F4 had been immobilized by amine-coupling chemistry. A marked signal was observed when the PG14 PrP-containing fraction was flowed onto the 15B3-coated chip, whereas no signal was observed with the *Prnp* ^0/0^ sample (Fig. 2A). The 15B3-bound PG14 fraction showed a very slow dissociation rate (4×10^−5^ s^−1^, Fig. 2A), suggesting a high-affinity interaction. No SPR signal was detected when the same P3 fractions were flowed onto sensor chips coated with antibody 3F4 (Fig. 2B), consistent with previous observations that the 3F4 epitope is inaccessible in native PG14 PrP molecules [18], [19]. These results indicated that 15B3 selectively bound aggregated PG14 PrP with high affinity, and could be used to immunopurify the protein.

### Immunopurification of Pathological PrP Aggregates Using Monoclonal Antibody 15B3

**Figure 3:**
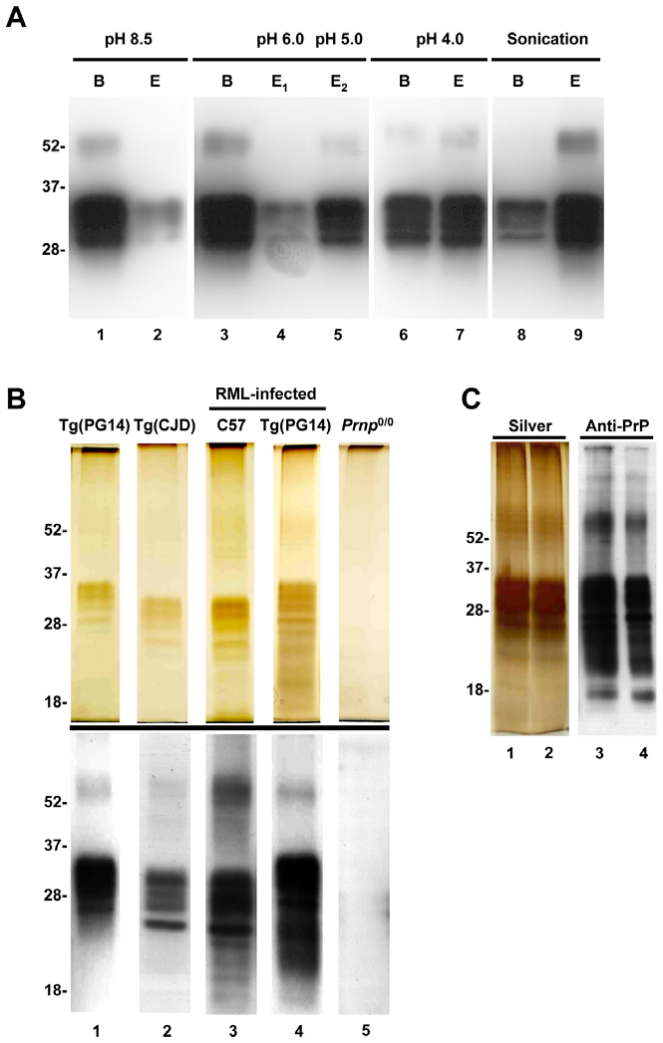
15B3 immunoprecipitation yields pure preparations of infectious and non-infectious mouse PrP aggregates. (A) PrP aggregates were immunoprecipitated from P3 fractions of Tg(PG14) brains with antibody 15B3. PG14 PrP eluted (E lanes) by incubation in 0.1 M Tris-Glycine buffer at different pH (lanes 1–7) or by sonication in PBS (lanes 8–9), and the residual protein eluted by boiling the Dynabeads in SDS (B lanes), were detected by Western blotting with anti-PrP antibody 3F4. (B) PrP aggregates, immunopurified and eluted by sonication, from un-inoculated Tg(PG14) (lane 1) and Tg(CJD) (lane 2) mice, or RML-infected C57BL/6 (lane 3) and Tg(PG14) (lane 4) mice were visualized by silver staining (top panel) or by Western blotting (bottom panel) using anti-PrP antibody 8H4. No protein bands were detected when the purification was done using *Prnp* ^0/0^ mouse brains (lane 5). (C) Two samples of PrP^Sc^ immunopurified from RML-infected C57BL/6 mice were run on SDS-PAGE, and the gels stained to saturation with silver (lanes 1–2) or immunoblotted with antibody 8H4 (lanes 3–4).

The P3 fraction from Tg(PG14) brains was immunoprecipitated with antibody 15B3, and several procedures were tested to elute PrP from the antibody without denaturing the protein. The antigen-antibody binding is usually disrupted by basic (>9.0) or acidic (<3.0) pH. Because these conditions may result in partial protein denaturation, we used solutions with a pH between 8.5 and 4.0. Negligible amounts of PrP were eluted at pH 8.5 and 6.0 (Fig. 3A, lanes 2 and 4), but recovery reached a maximum of ∼50% on lowering the pH to 5.0 or 4.0 (lanes 5 and 7).

We then tested procedures to improve PrP recovery. Subjecting the magnetic beads to three sequential rounds of sonication in phosphate-buffered saline (PBS) eluted more than 80% of immunoprecipitated PG14 PrP (Fig. 3A, lane 9). The eluted protein retained its aggregated state, as it partitioned in the pellet fraction after ultracentrifugation at 180,000×* g*, and was still recognized by 15B3 in the SPR or immunoprecipitation assay format (data not shown). SPR using known concentrations of immunopurified PG14 PrP enabled us to estimate an equilibrium dissociation constant (*K*_d_) of 1–2 nM monomer equivalent. However, since PG14 PrP is aggregated the actual affinity would be higher, depending on the size of the aggregates (e.g. *K*_d_ = 0.1−0.2 nM for decamers).

To assess the purity of the 15B3-immunoprecipitated aggregates, purified fractions from uninoculate or RML-inoculated Tg(PG14) mice, Tg(CJD) mice expressing the mouse homologue of the D178N/V129 PrP mutation linked to Creutzfeldt-Jakob disease (CJD) [27], and RML-infected C57BL/6 mice were subjected to SDS-PAGE and analyzed by silver staining or Western blot using anti-PrP antibodies (Fig. 3B and C). The majority of the immunopurified protein bands were in the expected M_r_ between 20 and 40 kDa (Fig. 3B, top panel), and reacted with antibody 8H4, whose epitope is located in the C-terminal region of PrP between residues 145 and 220 [28] (lower panel). A faint band with an apparent M_r_ of ∼60 kDa was also detected by 8H4 (Fig. 3B, lower panel, and Fig. 3C, lanes 3 and 4). This band could be clearly seen in silver-stained gels after longer exposure (Fig. 3C, lanes 1 and 2), and probably corresponded to SDS-resistant PrP dimers previously observed in prion-infected tissues [29]. Several PrP bands of low M_r_ were observed only in purified PrP^Sc^ preparations from RML-infected mice (Fig. 3B, lanes 3 and 4, and Fig. 3C). These bands did not react with an antibody that recognizes mouse PrP sequence 45–66 (not shown) [30], suggesting that they corresponded to N-terminally truncated PrP generated by proteolysis of PrP^Sc^ in infected brains [19], [31]. No proteins were detected in immunopurified samples from *Prnp* ^0/0^ mice (Fig. 3B, lane 5), confirming that the 15B3 antibody did not bind other insoluble proteins in the P3 fraction. To further assess the purity of the immunopurified preparations, we used MALDI-TOF mass spectrometry, and found that the only identifiable protein was PrP (probability-based MOWSE Mascot score: 85; matched peptides: 13; sequence coverage: 30%).

Comparative densitometric analysis of immunopurified PrP with serial dilutions of recombinant PrP as a standard (not shown) indicated a protein recovery rate of ∼1 µg per mouse brain.

### 15B3-Purified PrP^Sc^ Aggregates Seed Misfolding of PrP^C^

**Figure 4:**
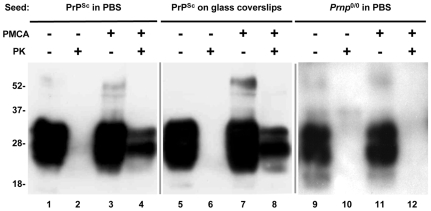
Immunopurified aggregates of PrP^Sc^ support *in vitro* misfolding of PrP^C^ by PMCA. Aggregates of PrP^Sc^ immunopurified from P3 fractions of RML-infected C57BL/6 mice (lanes 1–8), or immunoprecipitates of P3 fractions of *Prnp* ^0/0^ mice (lanes 9–12) were resuspended in PBS (lanes 1–4 and 9–12) or immobilized on glass coverslips by ultracentrifugation (lanes 5–8), and mixed with brain homogenates of Tg(WT) mice overexpressing 3F4-tagged PrP^C^. Mixtures were either rapidly frozen (lanes 1 and 2, 5 and 6, 9 and 10) or subjected to 90 cycles of sonication/incubation (lanes 3 and 4, 7 and 8, 11 and 12). Samples were then either subjected to proteinase K (PK) (lanes 2, 4, 6, 8, 10 and 12), or left undigested (lanes 1, 3, 5, 7, 9 and 11). All samples were analyzed by Western blotting with 3F4 antibody.

To test whether immunopurified PrP^Sc^ aggregates were able to seed misfolding of PrP^C^ we used PMCA technology [3], [32], [33]. A brain homogenate from Tg(WT) mice expressing 3F4-tagged mouse PrP^C^ [16] was seeded with PrP^Sc^ immunopurified from RML-infected C57BL/6 mice (which does not contain the 3F4 epitope), and the mixture was subjected to 90 cycles of sonication and incubation at 37°C. After PK digestion to eliminate the residual PrP^C^ substrate, the reactions were analyzed by Western blotting using antibody 3F4 to detect only newly formed protease-resistant PrP. Purified PrP^Sc^ aggregates, either in solution or immobilized on glass coverslips, seeded the formation of PK-resistant PrP (Fig. 4, lanes 4 and 8). Control PMCA reactions without sonication (Fig. 4, lanes 2 and 6), or seeded with immunoprecipitates from *Prnp* ^0/0^ mice (lanes 9–12), did not produce any protease-resistant PrP. These results indicated that immunopurified PrP^Sc^ was able to propagate its conformation *in vitro*.

### Mutant PrP Aggregates Are Toxic to Cultured Neurons

**Figure 5:**
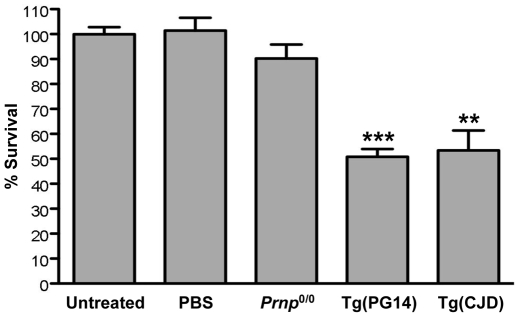
Mutant PrP aggregates from Tg(PG14) and Tg(CJD) mice are toxic to primary neurons. Cortical neurons from C57BL/6 mice were exposed to ∼15 nM of PrP aggregates purified from Tg(PG14) and Tg(CJD) mice, to the vehicle alone (PBS), or to 15B3-immunoprecipitated P3 fractions from *Prnp* ^0/0^ mice. Cell survival was quantified by MTT assay after seven days, and expressed as a percentage of untreated cells. Data are the mean±SEM of 8–24 replicates from three independent experiments. **p<0.005 and ***p<0.0001 *vs.* untreated (Mann-Whitney test).

There is evidence that purified preparations of PrP27-30 are toxic to cultured neurons [34]–[36]. We tested the effect of immunopurified PrP aggregates on the viability of cortical neurons cultured from newborn C57BL/6 mice. Cells were exposed to PrP aggregates (∼15 nM) isolated from the brains of Tg(PG14) or Tg(CJD) mice, and neuronal viability was assayed after seven days. There was a statistically significant toxic effect (Fig. 5). Neuronal viability approximately halved in aggregate-treated cells, whereas no toxicity was observed when cells were exposed to the vehicle alone (PBS), or to 15B3-immunoprecipitated fractions from *Prnp* ^0/0^ mice.

## Discussion

We have developed a protocol for purifying pathological PrP aggregates based on immunoprecipitation with a monoclonal antibody that recognizes structural features common to infectious and non-infectious forms of misfolded PrP. This method yields electrophoretically homogeneous PrP preparations without the need for protease digestion, and can be used to purify full-length PrP^Sc^ molecules, as well as other misfolded forms of the protein that lack infectivity and protease-resistance. The purified molecules retain their native biological properties, including the ability to seed misfolding of PrP^C^ by PMCA and neurotoxicity, and are suitable for structural and physicochemical studies.

Standard protocols for isolating PrP^Sc^ involve sequential centrifugation of detergent brain extracts to concentrate insoluble molecules, and PK digestion to eliminate PrP^C^ and other contaminating proteins, and enrich protease-resistant PrP. These methods are commonly used to purify PrP^Sc^ from prion-infected Syrian hamsters (typically inoculated with the 263K strain), which accumulate high levels of insoluble and protease-resistant PrP in their brains. The purity of PrP^Sc^ obtained with these procedures varies between 60 and 90%, depending on the characteristics of the prion strain, stage of disease of the animal, and the conditions in which the brains have been collected and stored [22]. Although PK readily digests most detergent-extracted proteins, ferritin can withstand even harsh PK treatment and often contaminates the final PrP^Sc^ preparation [37]. Moreover, due to protease treatment, the purified PrP^Sc^ protein lacks 65–70 aminoacids of the N-terminus. Hamster PrP^Sc^ has also been purified by immunoaffinity chromatography [21]; however, this method used antibodies that recognize both PrP^C^ and PrP^Sc^, and required PK digestion to isolate protease-resistant PrP^Sc^.

The protocol described here offers at least two major advantages over previous procedures. First, it consistently yields highly pure PrP preparations, independently of the amount of insoluble and protease-resistant PrP in the starting material. Second, since it does not require protease digestion, it can be used to purify different kinds of pathological PrP, including full-length PrP^Sc^ molecules, as well as other abnormally folded forms that lack protease resistance.

**Figure 6:**
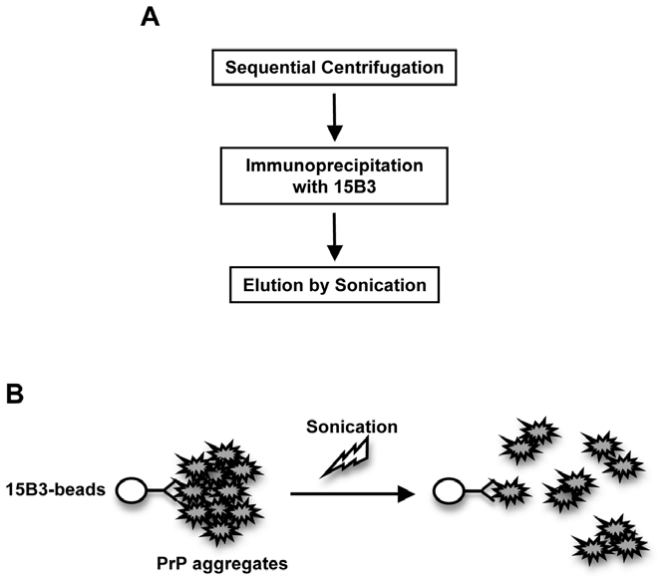
Diagram of the 15B3-immunopurification procedure. The scheme summarizes the main steps of the purification protocol (A), and illustrates the putative mechanism by which sonication elutes aggregated PrP from 15B3-coated beads (B).

The immunopurification method is straightforward and reproducible. Similarly to other procedures it requires a series of centrifugations to enrich insoluble PrP, then a single immunoprecipitation step with monoclonal antibody 15B3 (Fig. 6A). This antibody recognizes a variety of misfolded and aggregated forms of PrP from a wide range of species, including hamster, mouse, bovine and human [23]–[25]. Immunoprecipitated PrP is efficiently eluted by sonication, which probably breaks up the aggregates into smaller units, without disrupting the antigen-antibody binding (Fig. 6B). The eluted aggregates retain reactivity to antibody 15B3, and the ability to seed misfolding of PrP^C^ by PMCA, indicating that the structural feature that determine the 15B3 epitope and the converting activity of the purified protein are preserved. In addition, the immunopurified aggregates are toxic to cultured neurons, similar to PrP^Sc^ from infected hamsters [34]–[36], and β-folded oligomers of synthetic or recombinant PrP [38]–[43].

We show that 15B3 immunoprecipitation can be used to purify aggregated forms of mutant PrP that accumulate in the brains of spontaneously ill or RML-infected Tg(PG14) mice. In previous studies we analyzed PrP in brain homogenates of these mice using a panel of biochemical assays to try and identify structural differences between non-infectious and infectious forms of the protein. Surprisingly, we found that despite their marked difference in protease resistance both forms of PG14 PrP behave identically in a variety of biochemical tests for protein conformation and oligomeric state [19]. These results suggest that more subtle molecular differences distinguish infectious from non-infectious PrP. The nature of these differences can now be investigated using the immunopurified material described here which, for example, can be analyzed by atomic force microscopy, Fourier-transformed infrared spectroscopy, and mass spectrometry (our preliminary observations).

In conclusion, we have developed a procedure for obtaining pure preparations of aggregated PrP from different mouse models of prion disease, even those that accumulate abnormal forms of PrP that lack infectivity and/or protease resistance. Comparative analysis of purified PrP aggregates with different biochemical and biological properties could provide important information on their molecular nature and role in pathology.

## Materials and Methods

### Mice

All procedures involving animals and their care were conducted according to European Union (EEC Council Directive 86/609, OJ L 358,1; 12 December 1987) and Italian (D.L. n.116, G.U. suppl. 40, 18 February 1992) laws and policies, and in accordance with the United States Department of Agriculture Animal Welfare Act and the National Institute of Health (Bethesda, MA, USA) policy on Humane Care and Use of Laboratory Animals, and they were approved by the Animal Care Committee of the Department of Neuroscience at Mario Negri Institute (Milan, Italy).

Production of Tg mice expressing WT, PG14, or D177N/V128 mouse PrP tagged with an epitope for the monoclonal antibody 3F4 has been reported elsewhere [16], [27]. These Tg mice were originally generated on a C57BL/6J X CBA/J hybrid background, and were subsequently bred with the Zurich I line of *Prnp* ^0/0^ mice [44]. The resulting animals therefore express transgenically encoded but not endogenous mouse PrP.

For studies involving prion-infected mice, the RML isolate of scrapie (obtained from Byron Caughey and Richard Race, Rocky Mountain Laboratories, Hamilton, MT, USA) was passaged repeatedly in CD1 (Swiss) mice. Ten-percent (w/v) homogenates of mouse brain were prepared using a glass/Teflon apparatus (10 strokes at 1000 rpm) in ice-cold PBS. After being cleared by centrifugation at 900× *g* for 5 min, the homogenates were diluted to 1% in PBS, and 25 µl was injected intracerebrally into the right parietal lobe of weanling mice using a 25-gauge needle.

### Primary Neuronal Cultures

Cortical neurons were prepared from two-day-old C57BL/6 mice as described [45]. Briefly, brain tissue was sliced into ∼1 mm pieces and incubated in 5.8 mM MgCl_2_, 0.5 mM CaCl_2_, 3.2 mM HEPES, 0.2 mM NaOH (pH 7.4, 292 mOsm), containing 1 mg/ml papain (Sigma) at 34°C for 30 min. Dissociated cells were plated at 70,000 cells/well on poly-D-lysine-coated (0.05 mg/ml) 96-well plates, and maintained in Neurobasal Basal Medium (Gibco) supplemented with B27 (Gibco), penicillin/streptomycin and glutamine 2 mM (Gibco), at 37°C in an atmosphere of 5% CO_2_, 95% air. Cell viability was assessed by measuring the level of 3-(4,5 dimethylthiazol-2-yl)-2,5-diphemyl tetrazolium bromide (MTT) to formazan, as described [40].

### Sequential Centrifugation

Mouse brain homogenates were fractionated as described [19]. Brains were cut into small pieces, washed with PBS, homogenized with 3 ml of 10% sarcosine in TEND (10 mM Tris-HCl pH 8, 1 mM EDTA, 130 mM NaCl, and 1 mM dithiothreitol) containing a protease inhibitors cocktail (Cat. N. 1-836-153; Roche, Indianapolis, IN, USA), then incubated on ice for 1 h and centrifuged at 22,000× *g* for 30 min at 4°C. The supernatant was incubated on ice, while the pellet was resuspended in 1 ml of 10% sarcosine in TEND, incubated for 1 h on ice, and centrifuged at 22,000× *g* for 30 min at 4°C. The pellet (P0) was collected while the two supernatants were combined and centrifuged at 150,000×* g* for 2.5 h at 4°C. The new supernatant (S0) was removed and collected for further analysis, while the pellet was rinsed with 50 µl of 100 mM NaCl, 1% sulfobetaine (SB) 3-14, TEND plus protease inhibitors, resuspended in 1 ml of the same buffer, and centrifuged at 180.000×* g* for 2 h at 20°C. The supernatant (S1) was removed and collected, and the pellet was rinsed with 50 µl of TMS (10 mM Tris-HCl pH 7.0, 5 mM MgCl2, and 100 mM NaCl) plus protease inhibitors, resuspended in 600 µl of the same buffer containing 100 µg/ml RNAse A and incubated for 2 h at 37°C. The sample was then incubated with 5 mM CaCl_2_, 20 µg/ml DNAse I for 2 h at 37°C. To stop the enzymatic digestion, EDTA was added to a final concentration of 20 mM, and the sample was mixed with an equal volume of TMS, 1% SB 3-14.

The sample was deposited gently on a 100-µl cushion of 1 M sucrose, 100 mM NaCl, 0.5% SB 3-14, and 10 mM Tris-HCl pH 7.4, and centrifuged at 180,000× *g* for 2 h at 4°C. The supernatant (S2) was collected and the pellet was rinsed with 50 µl of 0.5% SB 3-14, PBS, resuspended in 100 µl of the same buffer, subjected to 3×5 s pulses of bath sonication with a Bandelin Sonopuls Ultrasonicator (Amtrex Technologies, Saint-Laurent, QC, Canada) at 90% power, and centrifuged at 180.000× *g* for 15 min at 4°C. The final supernatant (S3) was collected and the pellet (P3) was resuspended in 100 µl of 0.5% SB 3-14, PBS.

### Immunoprecipitation with 15B3

A 500-µl aliquot of mouse anti-IgM Dynabeads (Dynal, Carlsbad, CA, USA) and 250 µg of mAb 15B3 (Prionics, Zurich, CH) [23] were diluted with 1 ml of PBS containing 0.1% of bovine serum albumin (BSA), incubated for 2 h, washed three times with 1 ml of PBS and resuspended in 500 µl of PBS; 100 µl of 15B3-coated Dynabeads were then added to the resuspended P3 fractions. Each sample was incubated on a rotating wheel for 24 h at 4°C, after which beads were washed once with 1 ml of Wash Buffer (Prionics) and twice more with PBS. Beads were resuspended in 500 µl of PBS and then subjected to 3×30 s pulses of sonication using a bath microsonicator at 50% of power (Model 3000; Misonix, Farmingdale, NY, USA) to elute immunoprecipitated PrP. In order to increase the concentration of the purified aggregates the sample was ultracentrifuged at 100,000×* g* for 15 min at 4°C, then resuspended in 100 µl of 0.5% SB 3-14, PBS.

### Western Blotting

PrP was detected in purified fractions by Western blotting. Each sample was diluted 1∶1 in 2X Laemmli sample buffer, heated at 95°C for 5 min, then resolved by sodium dodecyl sulfate polyacrylamide gel electrophoresis (SDS-PAGE). Proteins were electrophoretically transferred to poly-vinylidene fluoride (PVDF) membranes, and the membranes were blocked for 10 min in 5% (w/v) non-fat dry milk in Tris-buffered saline containing Tween 20. After incubation with appropriate primary and secondary antibodies, signals were revealed using enhanced chemiluminescence (Amersham Biosciences), and were visualized by a Biorad XRS image scanner. Anti-PrP antibodies 3F4 [46] or 8H4 [28] were used to develop Western blots, as indicated in the figure legends.

### Silver Staining of Purified Fractions

Two µl of purified aggregates was diluted in 10 µl of Laemmli sample buffer and 4% urea, heated at 95°C for 10 min, then resolved by SDS-PAGE using 12% polyacrylamide gels. Gels were stained with Silver Snap (Pierce, Rockford, IL, USA) following the manufacturer's instructions. Murine recombinant PrP 23–231, used for indirect quantification of purified PrP aggregates, was expressed and purified according to the procedure of Zahn et al. [47] and stored in 50 mM sodium acetate, pH 4.0.

### Protein Misfolding Cyclic Amplification (PMCA)

Principles and details of the automated version of PMCA have been described elsewhere [33]. In our experimental conditions, Tg(WT-E1) brains were used as the source of 3F4-tagged PrP^C^ substrate. Ten-percent (w/v) brain homogenates were prepared in conversion buffer (PBS containing NaCl 150 mM, 1.0% Triton X-100, 4 mM EDTA, plus EDTA-free protease inhibitors). The samples were clarified by centrifugation at 300× *g* for 30 s in a tabletop centrifuge. One µl of PrP^Sc^ aggregates purified from RML-inoculated C57BL/6 mice (which produce non-3F4 tagged PrP^Sc^) was directly mixed (1∶200) with Tg(WT-E1) brain homogenate and loaded into 0.2-ml PCR tubes.

In an alternative setting, 1 µl of PrP^Sc^ aggregates was immobilized on a glass coverslip by ultracentrifugation at 150,000× *g* for 1 h at 4°C, then used for seeding the PMCA reaction. Tubes were positioned on an adaptor placed on the plate holder of a microsonicator (Model 3000; Misonix). The sonicator was programmed to perform 90 cycles of 30 min incubation at 37°C followed by a 40 s pulse of sonication at 60% power. Tubes were incubated without shaking, immersed in the sonicator bath, and the entire microplate horn was kept inside an incubator at 37°C. After completion of the PMCA reaction, 10 µl of each sample was diluted in PK digestion buffer (Tris-HCl 50 mM pH 8.5, 150 mM NaCl) and incubated with 50 µg/ml of PK at 37°C for 1 h on an end-over-end rotator before Western blotting with anti-PrP antibody 3F4.

### Surface Plasmon Resonance

SPR studies were carried out using the ProteOn XPR36 Protein Interaction Array system (Bio-Rad) [48]. Antibodies 15B3 and 3F4 were covalently immobilized in two parallel strips of the same GLC sensor chip (Bio-Rad), using amine-coupling chemistry. The chip surfaces were activated for 5 min with a mixture of 1-[3-(dimethylamino)propyl]- 3-ethylcarbodiimide hydrochloride (EDC, 0.2 M) and sulfo-N-hydroxysuccinimide (sulfo-NHS 0.05 M) (Bio-Rad); this was followed by injection of 3F4 (30 µg/ml in sodium acetate, pH 5.0), or 15B3 (30 µg/ml in sodium acetate, pH 5.0), which flowed for 5 min at a rate of 30 µl/min. The remaining activated groups were blocked with a 5-min injection of 1 M ethanolamine. The amounts of 3F4 or 15B3 covalently immobilized onto the surface, expressed in Resonance Units (1 RU = 1 pg protein/mm^2^), were about 6000 and 9000 RU, respectively. A reference channel was prepared in parallel using the same activation/deactivation procedure but injecting the vehicle only.

After the immobilization procedure, the fluidic system of ProteOn is rotated by 90° to test in parallel up to six different analytes over the target surfaces. P3 fractions isolated from the brain of Tg(PG14) or *Prnp* ^0/0^ mice, or 200–400 ng of immunopurified PG14 PrP, were diluted in PBS containing 0.5% NP-40 and 0.5% sodium deoxycholate, sonicated for 30 s and injected at a rate of 30 µl/min for 5 min. Vehicle was always injected in parallel flow channels. All the analyses were done at 25°C. The sensorgrams (time course of the SPR signal in RU) were normalized to a baseline value of 0. The signals observed in the surfaces immobilizing 15B3 or 3F4 were corrected by subtracting the non-specific response observed in the empty reference surface. Parallel injections of vehicle alone served to correct for binding-independent responses (i.e. drift effects).

### MALDI-TOF Mass Spectrometry and Protein Identification

Peptide mass fingerprinting (PMF) was performed on a Bruker ReflexIII^TM^ MALDI-TOF mass spectrometer equipped with a SCOUT 384 multiprobe inlet and a 337-nm nitrogen laser using as matrix α-cyano-4-hydroxycinnamic acid (Bruker, Daltonics), as described [49]. Data were subjected to NCBInr databases searching using as programs Mascot (http://www.matrixscience.com) allowing up to 1 missed trypsin cleavage and a mass tolerance of ±0.1 Da. In Mascot program, probability-based MOWSE scores [50] greater than 61 were considered significant (*p*<0.05) searching *Mus musculus* sequences deposited in NCBInr.
